# Anterior lens capsule and epithelium thickness measurements using spectral-domain optical coherence tomography

**DOI:** 10.1186/s12886-017-0489-0

**Published:** 2017-06-19

**Authors:** Jing Dong, Yading Jia, Yaqin Zhang, Haining Zhang, Zhijie Jia, Suhua Zhang, Qiang Wu, Qingmao Hu, Tianqiao Zhang, Xiaogang Wang

**Affiliations:** 1 The First Hospital of Shanxi Medical University, Taiyuan, Shanxi People’s Republic of China; 2 Shanxi Eye Hospital, 100 Fudong Street, Taiyuan, 030002 Shanxi People’s Republic of China; 3 Affiliated Sixth People’s Hospital Shanghai Jiao Tong University, Shanghai, People’s Republic of China; 4 Shenzhen Institutes of Advanced Technology, Chinese Academy of Sciences, 1068 Xueyuan Boulevard, Shenzhen, 518055 China; 5 Key Laboratory of Human-Machine Intelligence Synergy Systems, 1068 Xueyuan Boulevard, Shenzhen, 518055 China; 6 Shenzhen College of Advanced Technology, University of Chinese Academy of Sciences, 1068 Xueyuan Boulevard, Shenzhen, 518055 China

**Keywords:** Anterior lens capsular complex, Pupillary diameter, Optical coherence tomography, Signal strength index

## Abstract

**Background:**

To investigate the anterior lens capsule and epithelium thickness (defined as anterior lens capsular complex: ALCC) in normal Chinese subjects using spectral-domian optical coherence tomography (SD-OCT) and examine the factors that may influence the ALCC, such as age, gender, pupil diameter (PD) and signal strength index (SSI).

**Methods:**

A prospective observational case series. One-hundred-thirty-four normal subjects (134 eyes) were included. The ALCCs were determined manually via SD-OCT. Using the pupil center as a reference position, the central ALCC (CALCC), nasal 1-mm ALCC (NALCC), temporal 1-mm ALCC (TALCC) and PD were measured manually.

**Results:**

The mean CALCC, NALCC and TALCC were 33 ± 6 μm, 36 ± 7 μm and 34 ± 6 μm, respectively. The NALCC was significantly thicker than the CALCC (*P* < .001) and TALCC (*P* < .001). Moreover, CALCC was significantly thinner than TALCC (*P* = 0.013). Age was positively correlated with the CALCC (*r* = 0.292, *P* < .001), NALCC (*r* = 0.400, *P* < .001) and TALCC (*r* = 0.521, *P* < .001). PD, gender and SSI were not significantly correlated with the three ALCC parameters.

**Conclusions:**

The SD-OCT can be used to demonstrate the ALCC thickness, and age is positively correlated with the ALCC in the central, nasal and temporal sides.

## Background

The avascular lens is nourished by the surrounding aqueous humor and vitreous body. The anterior lens capsule with the accompanying monolayer subcapsular epithelium represents the most important metabolic element of the crystalline lens [1, 2]. Normally, the capsule and epithalium allows the passage of molecules both into and out of the lens and keeps the lens clear. In some pathological conditions, such as Fuchs syndrome, pesudoexfoliation syndrome etc., the metabolic balance could be damaged. This may be observed by the measuring of the lens capsule and epithelium thickness. Ophthalmic imaging technologies, such as ultrasound biomicroscopy, fluorescein angiography, confocal microscopy, Scheimpflug photography and optical coherence tomography (OCT), have widespread applicability in Ophthalmology [3–6]. As a cross-sectional, three-dimensional, completely noninvasive, high resolution imaging modality, OCT provides more detailed information for the anterior segment of the eye. Using a femtosecond laser OCT system with a central wavelength of 780 nm and an axial resolution of 2.3 μm, the lens capsule thickness map across the entire field of view has been manually measured in two healthy eyes of two volunteers [7]. The spectral domain OCT system used in present study provides 5 μm axial resolution, which is not capable of clearly demonstrating the border between the lens capsule and the subcapsular epithelium. However, it demonstrates a substantially more clear delineation when the highly reflective layers (the lens capsule and the subcapsular epithelium) are combined, which has mentioned that the tomogram correlates well with histological images in previous studies, and we defined as the anterior lens capsular complex (ALCC) in this study [7, 8].

The purpose of this prospective study was to measure the local ALCC thickness in normal Chinese subjects and to investigate potential factors that influence it, such as age, pupil diameter (PD), the signal strength index (SSI) and gender.

## Methods

### Study population

The study included 134 normal subjects (72 males, 62 females). Written informed consent was obtained from each subject after they were provided with an explanation of the nature of the study. For subjects with an age less than 18 years, written informed consent was obtained from their legal guardian. One eye from each subject was randomly selected for analysis. Han-Chinese individuals account for more than 90% of the Chinese population; thus, Han-Chinese subjects were selected using the unique ethnicity information on the volunteers’ identity cards. This approach helped to eliminate a potential influence from different ethnic groups. This study was performed at Shanxi Eye Hospital (Taiyuan, Shanxi, China). The research protocols were approved by the institutional review boards at Shanxi Eye Hospital and were conducted in accordance with the tenets of the Declaration of Helsinki. The subjects’ ages ranged from 5 to 86 years (mean, 49 ± 23 years). Eligible subjects had a normal ophthalmic examination that included the following: a best-corrected visual acuity of ≥20/40, a refractive error < 5 Diopter (D) sphere and <3D cylinder, normal slit-lamp and fundoscopy examinations, an axial length < 24.5 mm and an intraocular pressure < 22 mmHg. The exclusion criteria included any systemic disease, such as diabetes, hypertension, rheumatism etc., and all other detectable ocular diseases, a cortical cataract, recent ocular surgery, the use of contact lens, and the use of eye drops.

### OCT image processing

**Fig. 1:**
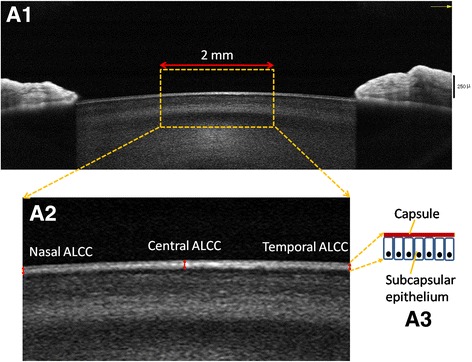
A horizontal scanning image of the central anterior part of the lens and the pupillary margin of the iris in a 29 year old male using spectral domain optical coherence tomography (**A1**). **A2** shows a magnification of the selected central 2-mm length. **A3** shows the schematic diagram of the anterior lens capsule and the subcapsular epithelium (corresponding to the two highly reflective layers between the *red arrows*), which we defined as the anterior lens capsular complex (ALCC). Using the pupil center as a reference position, the central ALCC, nasal 1-mm ALCC, temporal 1-mm ALCC were measured

Using an anterior segment, high resolution, cross line scan protocol, cross-sectional images of the iris and lens were captured via an Avanti RTVue XR spectral domain OCT (Optovue, Inc. USA) and a supplemental Cornea-Anterior Module (CAM) attachment in this study. The ALCC measurement, which is defined as the combination of the lens capsule and the corresponding subcapsular epithelium thickness (Fig. 1), was manually performed three times after image magnification by the same operator in each eye using internal measurement tool, and the results were averaged for further analysis. The Avanti RTVue XR has a scan length of 8 mm and a depth resolution of 5 μm for this anterior segment scanning. The light source of the system uses super luminescent diodes with a wave length of 840 nm and a scan rate of 70,000 A-scan/s. All images in this study were captured by an experienced and trained technician, and the image quality score for each image was greater than 15, which is good for measurement (software version 1.4.3.10). During each scan, the technician captured cross-sectional lens image with the light beam at the midpoint to ensure that the scan location was located in the center of the pupil. Using the pupil center as a reference position, the central ALCC (CALCC), nasal 1-mm ALCC (NALCC), temporal 1-mm ALCC (TALCC) and PD were measured.

### Inter-operator and inter-session reproducibility

The inter-operator reproducibility of the ALCC measurements was calculated from 10 subjects within a single visit by two operators (XG.W. and J.D.). The inter-session reproducibility of the ALCC was calculated from 10 subjects with 2 sets of scans obtained on 2 separate visits by the same operator (XG.W.).

### Statistics

Statistical analyses were performed with commercial software (SPSS ver. 13.0; SPSS, Inc.). The intraclass correlation coefficients (ICCs) were calculated to assess the inter-operator and inter-session reproducibility of the ALCC measurements. To compare the CALCC, NALCC and TALCC, paired t-tests were performed. A general linear model and a Pearson correlation analysis were used to evaluate the relationships between age, gender, PD, SSI and the ALCC. All tests had a significance level of 5%.

## Results

One hundred thirty-four subjects (134 eyes) were evaluated using the Avanti RTVue XR OCT system. The mean CALCC, NALCC and TALCC were 33 ± 6 μm, 36 ± 7 μm and 34 ± 6 μm, respectively. The NALCC exhibited significantly thicker values than the CALCC (*P* < 0.001) and TALCC (*P* < 0.001). Moreover, CALCC was significantly thinner than TALCC (*P* = 0.013). The mean PD and SSI were 6.02 ± 1.31 mm (range: 2.81–8.75 mm) and 45 ± 15 (range: 15–83), respectively.

**Fig. 2:**
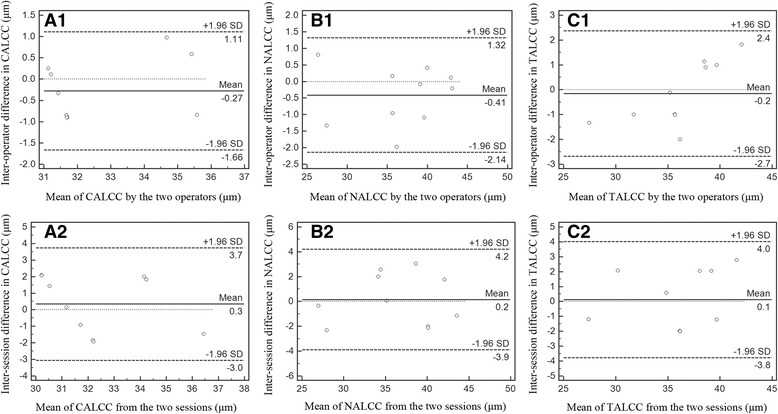
Graph of data from inter-operator (**A1**, **B1**, **C1**) and inter-session (**A2**, **B2**, **C2**) reproducibility study (*n* = 10). CALCC = central anterior lens capsular complex; NALCC = nasal anterior lens capsular complex; TALCC = temporal anterior lens capsular complex

In 10 randomly chosen subjects, the overall average CALCC, NALCC and TALCC were measured by operators, XG.W and J.D (Table 1). Table 2 shows the intraclass correlation coefficient (ICC) for the inter-operator and inter-session reproducibility; all values were approximately 0.9 with the exception of the CALCC from two visits (ICC = 0.712). Figure 2 (A1, B1, C1) shows a graph of the inter-operator differences against the means of the average CALCC, NALCC and TALCC. The 95% limits of agreement (LoA), defined as the mean interoperator difference in the CALCC, NALCC, TALCC ± (1.96 × SD of difference), are shown in each graph. The inter-session reproducibility was investigated using a similar approach. The graph of the differences against the mean for the inter-session data (Fig. 2: A2, B2, C2) indicates that all values fall within 1.96 SDs of the mean.

**Table 1:** Central, nasal and temporal anterior lens capsular complex of different measurements

	Measurements, Mean ± SD (*n* = 10)		
	Measurement 1	Measurement 2	Measurement 3
CALCC (μm)	32 ± 6	32 ± 7	33 ± 6
NALCC (μm)	36 ± 6	36 ± 7	37 ± 6
TALCC (μm)	36 ± 7	36 ± 7	36 ± 6

Measurements 1 and 2 indicate the 2 measurements performed by observer XG.W. on two sessions; measurement 3 comprises the third measurement performed by observer J.D. *CALCC* central anterior lens capsular complex, *NALCC* nasal anterior lens capsular complex, *SD* standard deviation, *TALCC* temporal anterior lens capsular complex

**Table 2:** Inter-operator and inter-session reproducibility analysis of the central, nasal and temporal anterior lens capsular complex

	Intraclass correlation coefficient	95% confidence interval	
		Lower bound	Upper bound
CALCC 1–2	0.712	0.223	0.919
CALCC 1–3	0.927	0.737	0.981
NALCC 1–2	0.941	0.795	0.985
NALCC 1–3	0.988	0.954	0.997
TALCC 1–2	0.909	0.694	0.976
TALCC 1–3	0.953	0.824	0.988

Numbers 1 and 2 indicate the 2 measurements performed by observer XG.W. on two sessions; measurement 3 indicates the third measurement performed by observer J.D. *CALCC* central anterior lens capsular complex, *NALCC* nasal anterior lens capsular complex, *TALCC* temporal anterior lens capsular complex

**Fig. 3:**
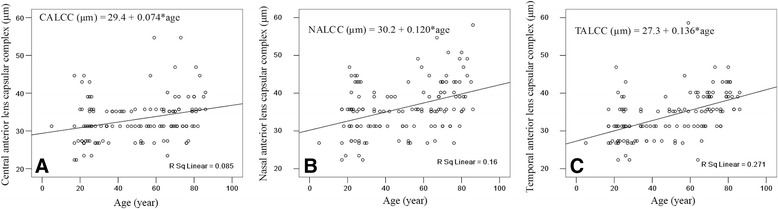
Scatter plot of (**a**) age against the central anterior lens capsular complex, (**b**) age against the nasal lens capsular complex, and (**c**) age against the temporal anterior lens capsular complex as measured by spectral domain optical coherence tomography. Line: univariate regression summarizes the relationship between the two variables. CALCC = central anterior lens capsular complex; NALCC = nasal anterior lens capsular complex; TALCC = temporal anterior lens capsular complex

Figure 3a shows the regression of the CALCC on age. A linear model demonstrated this relationship: CALCC (μm) = 29.4 + 0.074*age (*r* = 0.292, *P* < .001). Figure 3b shows the regression of the NALCC with age. A linear model demonstrated this relationship: NALCC (μm) = 30.2 + 0.120*age (*r* = 0.400, *P* < .001). Figure 3c shows the regression of the TALCC on age. A linear model demonstrated this relationship: TALCC (μm) = 27.3 + 0.136*age (*r* = 0.521, *P* < .001). According to the previously described models, a 10-year increase in age results in an approximate 0.74, 1.2, and 1.4 μm increase in the CALCC, NALCC and TALCC, respectively. PD, gender and SSI were not significantly correlated with the three ALCC values (Table 3).

**Table 3:** Correlations between gender, age, pupil diameter, or signal strength index and the anterior lens capsular complex thickness

	Central ALCC	Nasal ALCC (1-mm)	Temporal ALCC (1-mm)
Gender	−0.067 (0.443)	0.122 (0.162)	−0.003 (0.969)
Age	**0.292 (0.001)**	**0.400 (.000)**	**0.521 (.000)**
PD	0.026 (0.770)	0.049 (0.573)	0.161 (0.063)
SSI	0.080 (0.355)	0.073 (0.401)	−0.049 (0.571)

*ALCC* anterior lens capsular complex, *PD* pupil diameter, *SSI* signal strength index. All values calculated by Pearson correlation analysis. Numbers indicate the Pearson correlation coefficient (*P* values). Significant correlation coefficients (*P* < 0.05) are in bold

## Discussion

As a result of the limited axial resolution and scanning speed, current commercial imaging technologies, such as ultrasound biomicroscopy, a Scheimpflug camera, the time-domain OCT of 1310 nm wavelength and the spectral domain OCT at approximately 830 nm wavelength, fail to provide accurate cross-sectional estimation of the complete lens capsular bag, especially the independent evaluation of the lens capsule or subcapsular epithelium measurements in vivo [9, 10]. However, it is possible to determine the ALCC thickness based on the good correlation between the tomogram and the histological images [7]. Therefore, we measured the ALCC thickness in for subjects from 5 years to 86 years of age to obtain broader anatomical evidence for the human lens structure using OCT in vivo.

The present study indicates that the central, nasal and temporal ALCC were reproducible using a commercial, ultrahigh resolution OCT. Compared with the high inter-operator ICCs, relatively lower ICCs were identified for the inter-session reproducibility analysis. The high inter-operator ICCs indicate that the operator is not a significantly influential factor for the measurement, especially for professional technicians or ophthalmologists. The relatively lower inter-session ICCs may be attributed to the fluctuation of the cross-sectional lens structure image capture on different visit. The average thickness of the CALCC was approximately 33 μm, which is approximately 15 μm thicker than the lens capsule values identified during previous OCT and other imaging technologies [7, 11, 12]. The ALCC comprises a combination of the anterior lens capsule and a monolayer of epithelial cells; thus, we hypothesized that the lens epithelium thickness is approximately 15 μm in vivo.

For the age-related cataract eyes, we excluded the cortical cataract, which may cause dysfunction of the active transportation of electrolytes because of capsular epithelium breakdown [1]. The NALCC was significantly thicker than the CALCC and TALCC. Significant difference was also identified between the CALCC and TALCC in this study. This difference may be attributed to the potentially asymmetry of the lens structure, especially in the accommodative situation.

The PD ranged from 2.81 to 8.75 mm; however, there was no statistical correlation with the three ALCC values. This finding may be because the lens capsule is primarily composed of Type IV collagen, and it is sufficiently elastic to handle the accommodation range in this study. Although female gender was correlated with an increased rate of epithelial cell proliferation, no significant correlation was identified between gender and the ALCC in this study [13]. This finding may be because we only included normal subjects in the current study. Previous research has demonstrated that there is no significant lens thickness difference between normal female and male subjects using anterior segment OCT, which may also partially support our findings [14]. An increased SSI yield improved the segmentation and image clarity. The SSI was not significantly correlated with the ALCC values in this study. This finding may be because the ALCC measurement boundaries include a relatively hyperreflective band and the normal ocular median, such as the cornea and aqueous humor, which minimally affects the measurement procedure in this study.

Previous studies have demonstrated that both the lens capsule thickness and lens thickness were positively correlated with age [14, 15]. These findings were consistent with our findings regarding the positive correlations between age and the ALCC thickness.

ALCC plays an important role in allowing the passage of molecules into and out of the lens, which is vital for keeping lens metabolic balance. ALCC measurements in vivo using a current commercial OCT system provide substantial help for clinical and scientific applications. It may substantially facilitate studies regarding accommodation, presbyopia, posterior capsule opacification, Fuchs syndrome and pesudoexfoliation syndrome, as well as the assessment of anterior lens capsule complex related cataract surgery risk factors, especially for mature cataract [11, 16–18].

The OCT system used in this study for ALCC imaging has several limitations. First, the automatic real-time technology, which improves the image visualization quality using frame averaging or de-speckling in this study, may occasionally cause distorted or blurred structure delineation because of patient fixation fluctuation during image capture. Second, the axial resolution of the current 840 nm OCT system is not adequate to clearly demonstrate the lower boundary of the lens capsule. Therefore, we determined the ALCC thickness in this study. Third, the mirror-like reflections demonstrated in the center, which may be caused by the lens structure laying perpendicular to the light beam, may affect the manual measurements [15]. Therefore, measurements were performed three times by the same operator in each eye, and the averaged data were used for the final analysis to reduce this potential influence and to reduce the manual measurement variability. Finally, we only observed the central 2-mm diameter, which is not sufficient to demonstrate the whole lens structure changing tendency; this limitation may be addressed in the future using a substantially higher axial resolution and higher scanning speed OCT system or other imaging technologies.

## Conclusion

In conclusion, this study demonstrated that commercial spectral domain OCT may be used to determine the ALCC thickness and that age is positively correlated with the ALCC.

## Abbreviations

ALCC: Anterior lens capsular complex
CALCC: Central anterior lens capsular complex
D: Diopter
ICCs: Intraclass correlation coefficients
NALCC: Nasal anterior lens capsular complex
PD: Pupil diameter
SD: Standard deviation
SD-OCT: Spectral-domian optical coherence tomography
SSI: Signal strength index
TALCC: Temporal anterior lens capsular complex
